# A Temperature-Dependent Model for Tritrophic Interactions Involving Tea Plants, Tea Green Leafhoppers and Natural Enemies

**DOI:** 10.3390/insects13080686

**Published:** 2022-07-29

**Authors:** Huaguang Qin, Wuxuan Hong, Zehua Qi, Yinghong Hu, Rui Shi, Shuyuan Wang, Yuxi Wang, Jianping Zhou, Dan Mu, Jianyu Fu, Tingzhe Sun

**Affiliations:** 1 The Province Key Laboratory of the Biodiversity Study and Ecology Conservation in Southwest Anhui, School of Life Sciences, Anqing Normal University, Anqing 246133, China; qhg@aqnu.edu.cn (H.Q.); m15156017323@163.com (W.H.); qizehua77@163.com (Z.Q.); huyinghong91092002@163.com (Y.H.); shirui_1999@163.com (R.S.); wangshuyuan202103@163.com (S.W.); wangyuxi0027@163.com (Y.W.); mudansmile@126.com (D.M.); 2 Wanxinan Products Quality Supervision and Testing Center, Anqing 246052, China; zjpwhp@163.com; 3 Tea Research Institute, Chinese Academy of Agricultural Sciences, Hangzhou 310008, China

**Keywords:** tea plant, tea green leafhopper, tritrophic relationship, slow release, temperature dependency

## Abstract

**Simple Summary:**

Tritrophic interactions have achieved much attention in research on ecology. The tea plant *Camellia sinensis* (L.) O. Kuntze is a major economic crop in Asian countries, especially in China. Tea plants suffer infestations from herbivory attack during their lifetime. The tea green leafhopper (*Empoasca onukii* Matsuda) is a major pest for tea plants. The parasitic or predatory natural enemies of tea green leafhoppers (TGLs) can feed on their eggs, nymphs, or adults. However, a detailed mathematical model for tea plants–TGLs–natural enemies is still lacking. In the current work, we established a novel model based on laboratory measurements or field observations with temperature-dependent effects on tritrophic interactions for tea ecosystems. As expected, cyclic behaviors are identified. Stochastic simulations further showed two TGL outbreaks, the timing of which is consistent with field observations. Effective accumulated temperature (EAT) is possibly an important predictor of TGL outbreak. Applying slow-releasing semiochemicals as either repellents or attractants may be highly efficacious for pest biocontrol. An optimal treatment time of semiochemicals can also be determined. Our detailed model identifies key features of tritrophic interactions involving tea plants and can be extended to other ecosystems.

**Abstract:**

The tea green leaf hopper, *Empoasca onukii* Matsuda, is a severe pest of tea plants. Volatile emissions from tea shoots infested by the tea green leafhopper may directly repel insect feeding or attract natural enemies. Many studies have been conducted on various aspects of the tritrophic relationship involving tea plants, tea green leafhoppers and natural enemies. However, mathematic models which could explain the dynamic mechanisms of this tritrophic interaction are still lacking. In the current work, we constructed a realistic and stochastic model with temperature-dependent features to characterize the tritrophic interactions in the tea agroecosystem. Model outputs showed that two leafhopper outbreaks occur in a year, with their features being consistent with field observations. Simulations showed that daily average effective accumulated temperature (EAT) might be an important metric for outbreak prediction. We also showed that application of slow-releasing semiochemicals, as either repellents or attractants, may be highly efficacious for pest biocontrol and can significantly increase tea yields. Furthermore, the start date of applying semiochemicals can be optimized to effectively increase tea yields. The current model qualitatively characterizes key features of the tritrophic interactions and provides critical insight into pest control in tea ecosystems.

## 1. Introduction

The tea plant *Camellia sinensis* (L.) O. Kuntze is a major economic crop in Asian countries, and tea is also a non-alcoholic drink worldwide [1]. As with other crops, tea plants suffer infestations from herbivores during their lifetime. Specifically, the tea green leafhopper (TGL), *Empoasca onukii* Matsuda (Hemiptera: Cicadellidae), is probably the most serious constraint to tea plant cultivation [2]. Nymphs or adults of TGLs attack the tea plants by sucking the sap, and their outbreaks can significantly reduce tea yields and quality [3].

Plants have evolved diverse mechanisms against herbivore attack [4]. Herbivory attack induces the release of plant volatiles, termed as herbivore-induced plant volatiles (HIPVs) [4]. These HIPVs are involved in tea plant defense, either directly by repelling herbivores, or indirectly by attracting natural enemies of herbivores [5,6]. For example, two parasitic wasps, *Stethynium empoascae* Subba Rao and *Schizophragma parvula* Ogloblin can parasitize the eggs of tea green leafhoppers, with a parasitism rate of 40 to 65% [2,7,8]. Recent reports have shown that HIPVs induced by tea green leafhopper infestation in tea plants can attract these parasitic wasps, which serve as natural enemies of tea green leafhoppers [9,10]. This volatile-mediated tritrophic interaction between tea plants, tea green leafhoppers and parasitoid wasps has received much more attention in integrated pest management [11,12].

In the past few decades, interest in the design of mathematical models based on population dynamics to aid pest management has increased [13,14,15,16,17]. Most studies which focused on predator–prey interactions often assume that the environments are homogeneous [18,19]. The natural system, however, is inevitably influenced by various environmental factors (e.g., temperature). Temperature is a determinant factor and has significant impact on processes related to ecological community, particularly predator–prey or consumer–resource interactions [20,21]. For example, the growth rate of crops, the oviposition, and the lifespan of pests can be altered by temperature [22,23]. For predator–prey systems, variations in temperature usually have a nonlinear effect on the short-term interaction strength by regulating the foraging and attack rates of predators [24]. Furthermore, the thermal effect on prey traits is also relevant to the predator–prey interactions [25]. Many species, including insects, undergo ontogenetic shifts, either in the form of habitats or life cycles with distinct developmental stages [20]. This is critical, since the consumption of some prey species is dependent on the prey body size or its developmental stage [26]. Predator and prey traits may sometimes have asymmetric thermal responses; for instance, the optimal temperatures for foraging and development are different, or predator and prey traits respond differentially across a thermal gradient [20]. Under these circumstances, dynamic behaviors in tritrophic or interlocked predator–prey systems may become complicated owing to temperature-dependent effects.

Additionally, there is considerable evidence that noise or randomness can play an important role in ecological systems [27]. These noisy fluctuations can arise from either intrinsic sources, which are associated with inherent stochasticity in ecological models (e.g., randomness in prey selection), or from extrinsic sources, which correlate with environmental factors (e.g., temperature) [28]. As a result, deterministic models are imprecise for characterizing the dynamic evolution of tritrophic interactions in tea agroecosystems, and stochastic models should be used. However, to the best of our knowledge, a temperature-dependent stochastic model which characterizes the tritrophic interactions among tea plants, tea green leafhoppers, and natural enemies is still lacking. To facilitate better understanding of tritrophic interactions in tea ecosystems, we constructed a detailed and realistic stochastic model based on ordinary differential equations (ODEs) with variations in daily temperature and thermal effects on tea growth and developmental stages of tea green leafhoppers. Stochastic simulations identified two peaks or outbreaks of tea green leafhoppers in a year, with markable variations in the peaking time. The role of parasitoids or predators in the tritrophic interactions was evaluated. The results indicated that predators may be highly efficacious for leafhopper control compared with parasitoids. The relationship between daily average effective accumulated temperature (EAT) and tea green leafhopper outbreaks was also established. An optimum treatment using semiochemicals was also proposed to increase tea yields. Collectively, our model characterized the dynamic thermal responses of tritrophic relationship involving tea plants, tea green leafhoppers, and natural enemies to provide important insight into the development of optimum strategies for tea pest control.

## 2. Materials and Methods

### 2.1. Model Construction

**Figure 1:**
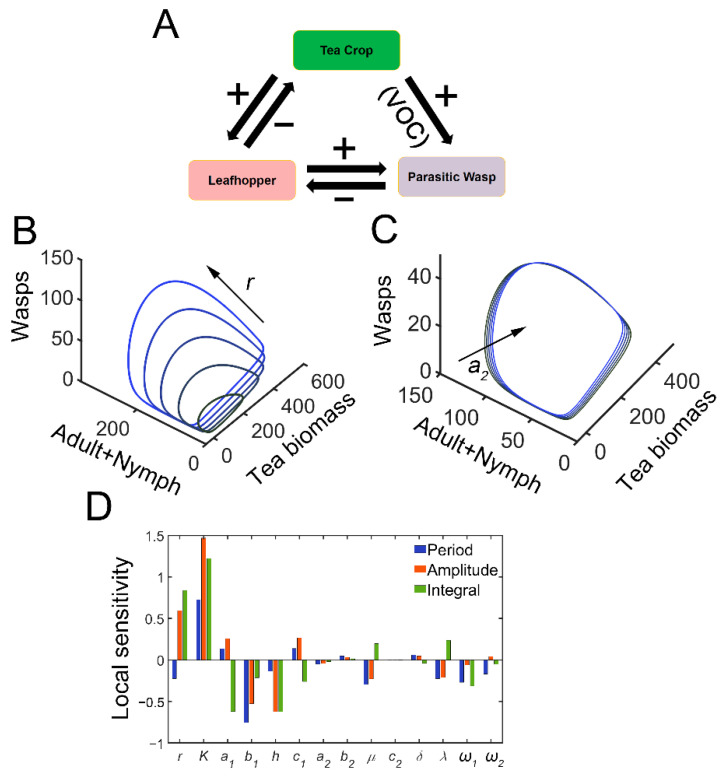
Tritrophic interactions exhibit sustained oscillations. (**A**) Schematic diagram of tritrophic relationship among tea crops, TGLs, and natural enemies (parasitic wasps). The feeding damage by TGLs induces the release of volatiles, which can attract the wasps. (**B**) Limit cycles in 3D plot with increasing growth rate of tea crops (*r*). In the simulation, *r* is increased from 4 to 12, with an increment of 2. (**C**) Limit cycles in 3D plot with increasing parasitic rate (*a*_2_). *a*_2_ is increased from 0.4 to 2, by 0.4. (**D**) Local sensitivity analysis for period (blue), amplitude (red), and integrated tea crop levels (green, integral, or tea yields).

We first constructed a tea plant–tea green leafhopper–natural enemy model, and the output was used to propose optimum biocontrol strategies. In model (1), *C* represents the tea crop population, whereas *P* and *W* denote adult tea green leafhoppers and parasitic wasps, respectively. *E* denotes leafhopper eggs and *N* represents the nymph population. Previously, Holling proposed Holling-type functional responses in the predator–prey system [29]. Recently, several authors have found that the Holling type II response can make the standard predator–prey model more realistic [30,31]. Therefore, we assumed that the consumption of adult leafhoppers or nymphs follows Holling type II functional responses [29]. *r* is the intrinsic growth rate of tea plants and *K* is the environmental capacity. Since the adults and nymphs of tea green leafhoppers can pierce and suck the sap of tender tea shoots [32], *P* and *N* can both feed on tea crops. *a*_1_ denotes the predation rate of pests. *a*_2_ indicates the parasitic rate. *h* is the harvesting or death rate of tea crops. *c*_1_ represents the conversion efficiency from adult leafhoppers to eggs by feeding on tea crops. Similarly, *c*_2_ is the conversion efficiency of parasitic wasps from the egg population. *b*_1_ and *b*_2_ are the half saturation rates for the feeding process. *μ* represents the natural death rate of pests. *δ* describes the dispersal rate for parasitic wasps (flying away from the sites of infestation). The parameters were generally adopted from the work by Mandal et al. with a few modifications to fit our tea plant–tea green leafhopper–natural enemy system or observations [31]. The feeding damage on the tea crop by leafhoppers induces the synthesis and emission of VOCs which can attract natural enemies (Figure 1A) [5,6]. *λ* characterizes the attraction rate of parasitic wasps induced by feeding damage. The developmental stages of leafhoppers include eggs, nymphs, and adults [33]. *ω*_1_ is the (average) conversion rate from eggs to nymphs, and *ω*_2_ is the (average) conversion rate from nymphs to adult leafhoppers. The following ODEs were developed based on the above assumptions. For parameter values and initial conditions, please refer to Table S1.

(1) $$\begin{array}{l} \frac{dC}{dt}=rC(1-\frac{C}{K})-\frac{a_{1}C(P+N)}{b_{1}+C}-hC\\ \frac{dP}{dt}=\omega_{2}N-\mu P\\ \frac{dW}{dt}=\frac{c_{2}a_{2}EW}{b_{2}+E}+\frac{\lambda a_{1}C(P+N)}{b_{1}+C}-\delta W\\ \frac{dE}{dt}=\frac{c_{1}a_{1}CP}{b_{1}+C}-\omega_{1}E-\frac{a_{2}EW}{b_{2}+E}\\ \frac{dN}{dt}=\omega_{1}E-\omega_{2}N \end{array}$$

### 2.2. Stochastic Tea Pest–Natural Enemies Model with Thermal Effect

The deterministic model only describes average population dynamics and may be imprecise if stochasticity is considered. We further constructed a realistic and stochastic model which incorporates the effect of daily temperature on tea crop growth, developmental stages, and lifespan of tea green leafhoppers. The effect of temperature on plant growth rate was formulated as previously described [34]. Briefly, the coefficient for temperature-driven effect (*TF*) can be expressed as:

(2) $$TF=e^{-{\left(\frac{T-T_{0}}{\beta }\right)}^{2}}$$

where *T*_0_ denotes the optimal temperature on tea growth. Following the work by Chen et al. [23], *T*_0_ was set to be 23 °C, and *β* was estimated to be 20 °C. The temperature-dependent tea growth rate can then be expressed as:

(3) r(T)=r⋅TF

The tea growth rate *r* was replaced by *r*(*T*) and modified in stochastic simulations. The egg, nymph, and pre-oviposition stages during tea green leafhopper development are also temperature-dependent [35]. The relationship between temperature and stage durations can be described using the Arrhenius equation $a\cdot e^{-bT}$ [20], where *T* is the temperature. The parameter fitting results are listed in Table S2 and Figure S1. Note that in this case, the logistic function was not statistically significant, as all fitted parameters had confidence intervals that contained zeros. The effect of temperature on mean lifespan of eggs, nymphs, and adults was also fitted using the Arrhenius equations. The fitting results are shown in Table S2.

According to the law of exponential decay, the kinetic parameters for degradation/conversion rates *σ* and the mean lifetime/duration of a stage *τ* can be interchanged using a reciprocal relation.

(4) $$\sigma =\frac{1}{\tau }$$

Therefore, *μ*, *ω*_1_, or *ω*_2_ can be regarded as degradation/conversion rates and are functions of temperature *T*. The parameters *μ*, *ω*_1_, or *ω*_2_ were replaced by *μ*(*T*), *ω*_1_(*T*), or *ω*_2_(*T*) with 10% variations. We did not differentiate the five instars during the nymph stage. The temporal duration of nymph and pre-oviposition stages were combined to calculate *ω*_2_(*T*), because leafhoppers at nymph and pre-oviposition stage can both feed on tea crops but are immature (unable to lay eggs). The threshold for the different developmental stages was also considered. The threshold temperatures determined for the egg and nymph (pre-oviposition) stages are approximately 11.06 °C and 7.86 °C, respectively [35]. Therefore, we obtained the following expressions:

(5) $$\begin{array}{l} \psi_{1}=\left\{ \begin{array}{ll} 1 & T\geq T_{\theta 1}\\ 0 & T<T_{\theta 1} \end{array}\right.\\ \psi_{2}=\left\{ \begin{array}{ll} 1 & T\geq T_{\theta 2}\\ 0 & T<T_{\theta 2} \end{array}\right. \end{array}$$

T*_θ_*_1_ is the developmental threshold temperature for eggs. *T_θ_*_1_ was set to be 11 °C in our model. *T_θ_*_2_ is the threshold temperature for nymphs. *T_θ_*_2_ was 8 °C in our model. The final equations for stochastic simulations are shown as follows:

(6) $$\begin{array}{l} \frac{dC}{dt}=r(T)C(1-\frac{C}{K})-\frac{a_{1}C(P+N)}{b_{1}+C}-hC\\ \frac{dP}{dt}=\psi_{2}\omega_{2}(T)N-\mu (T)P\\ \frac{dW}{dt}=\frac{c_{2}a_{2}EW}{b_{2}+E}+\frac{\lambda a_{1}C(P+N)}{b_{1}+C}-\delta W\\ \frac{dE}{dt}=\frac{c_{1}a_{1}CP}{b_{1}+C}-\psi_{1}\omega_{1}(T)E-\frac{a_{2}EW}{b_{2}+E}\\ \frac{dN}{dt}=\psi_{1}\omega_{1}(T)E-\psi_{2}\omega_{2}(T)N \end{array}$$

The temperature (*T*) varies over a year and, therefore, we obtained the average daily temperature at Anqing City in 2020 from the National Oceanic and Atmospheric Administration website (Figure S3, http://www.noaa.gov/, accessed on 5 May 2022).

### 2.3. Local Sensitivity Coefficient

Suppose a dynamic system ***x****’ = F*(***x****, **θ***), where ***x*** and ***θ*** are state and parameter vector, respectively. ***θ*** = (*θ_1_, θ_2_, …, θ_i_, …, θ_n_*), where *n* is the number of model parameters. For a certain feature ‘*I*’ (period, amplitude, or integral), its local sensitivity coefficient ***S*** with respect to changes in parameter *θ_i_* is defined as:

(7) S=∂II∂θiθi

When ‘*I*’ stands for integrated response or integral, it is defined as:

(8) $$I=\int_{0}^{T_{end}}A\cdot dt$$

where *T_end_* is the end of a year, and *A* denotes the level of certain population (tea crop, eggs, adult leafhoppers, or nymphs). The integrated response of tea crops is regarded as tea yields. In numerical simulations, the calculation of local sensitivities is obtained by increasing the parameter *θ_i_* by 1% and keeping all the other parameters fixed; we then get the perturbed response *I_p_* for feature ‘*I*’. *I_default_* is response for feature ‘*I*’ using default parameters (Table S1). Thus, in our model, $\partial I/I=\left(I_{p}-I_{default}\right)/I_{default}$, and ${\partial \theta_{i}/\theta }_{i}$ = 1%.

### 2.4. Stochastic Simulation

Stochastic simulation based on ODEs was implemented using a Poisson *τ*-leap method [36]. Briefly, suppose there are *N* populations in the system [*S_1_*, *S_2_*,…, *S_i_*, …, *S_N_*] with molecular number ***x*** = *X_i_*(*t*) for species *S_i_* at time *t*. The reaction channels (list of reactions) are represented as [*R_1_*, *R_2_*,…, *R_M_*], and *M* is the number of reactions. For the reaction channel *R_j_*, *a_j_*(***x***)d*t* denotes the probability that the reaction *R_j_* will occur in the time interval [*t*, *t +* d*t*). A state change vector ***ν****_j_* is defined to characterize reaction channel *R_j_*. *ν_ij_* is the element of ***ν****_j_* and represents the change in molecular numbers of population *S_i_* due to the reaction *R_j_*.

In the Poisson τ-leap method, we assume that there are many reactions firing in a relatively large time interval [*t*, *t + τ*) [36]. The number of reactions for channel *R_j_* firing at the interval [*t*, *t + τ*) obeys Poisson distribution with mean *a_j_*(***x***)*τ*. The system is updated by:

(9) $$x(t+\tau )=x(t)+\sum_{j=1}^{M}\nu_{j}P(a_{j}(x)\tau )$$

‘*P*’ denotes the Poisson distribution. The basic framework for implementation of this stochastic algorithm based on ordinary differential equations is provided in Supplementary Materials.

### 2.5. Model Simulation

The ordinary differential equations were integrated with the ode23s solver. Numerical and stochastic simulations were performed in MATLAB (MathWorks, Natick, MA, USA, R2018b).

## 3. Results

### 3.1. The Tea Plant–TGL–Wasp System Exhibits Sustained Oscillation

**Figure 2:**
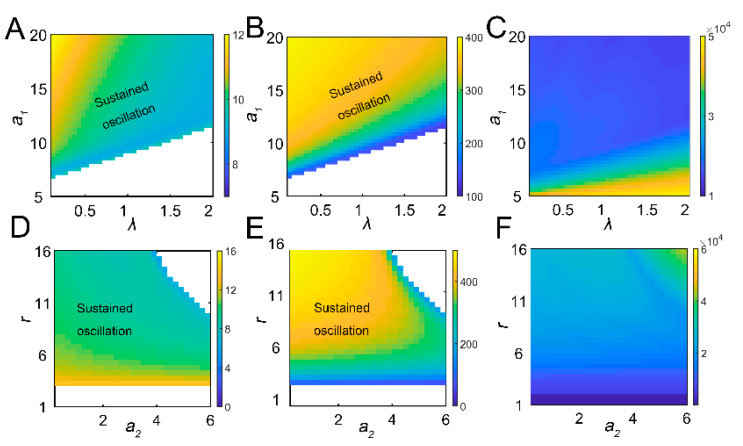
The global effect of parameters on dynamic responses. The combinatorial effects of *a*_1_ and *λ* on period (**A**), amplitude (**B**), and tea crop integral (**C**). The regions of parametric combinations leading to sustained oscillation were marked as “sustained oscillation”. The combinatorial effect of *a*_2_ and *r* on period (**D**), amplitude (**E**), and tea crop integral (**F**).

The tritrophic interactions among tea plants, TGLs, and parasitic wasps were formulated using model 1. After infestation, the feeding damage by TGLs induces the emission of volatiles from the tea crop, and the VOCs may attract natural enemies (dominant parasitoids *S. empoascae* or inferior species *S. parvula*) (Figure 1A). Using the default parameters, the system exhibited sustained oscillations, as evidenced by limit cycles (Figure 1B,C and Table S1). We noted the existence of interlocked positive (tea–parasitic wasps–leafhoppers) and negative (tea–leafhoppers and leafhoppers–parasitic wasps) feedback loops in the tritrophic system, which may potentially lead to limit cycle oscillations [37]. Indeed, a supercritical Hopf bifurcation can be identified (Figure S2). The attraction of natural enemies to the sites of herbivory attack by volatiles creates a positive feedback loop in the tritrophic interaction system (Figure 1A), and the positive feedback may function to stabilize the limit cycle oscillations [37]. Increasing the growth rate of tea plants enlarged the limit cycles (Figure 1B), whereas increasing the parasitic rate (*a*_2_) slightly shrank the size of the limit cycles (Figure 1C). Local sensitivity analysis showed that parameters for tea growth (*r* and *K*) negatively affected the period, whereas enhanced insect-feeding processes (*a*_1_ and *b*_1_) tended to elongate the period (Figure 1D). The process related to tea growth also increased the amplitude and temporal integral (tea yields) of tea plants (Figure 1D). TGL feeding indeed decreased the tea yields (Figure 1D). We then extended the parametric regions and evaluated the systemic responses. Undamped oscillations were allowed in broad regions for *a*_1_ and *λ* (Figure 2A,B). Interactions between *a*_1_ and *λ* were also identified. For relatively large *λ* values, the effect of *a*_1_ on oscillatory periods and amplitudes was weakened (Figure 2A,B). There was also an interaction between parameters *r* and *a*_2_, as we observed that the effect of *r* on oscillatory patterns vanished when the value of *a*_2_ was large (Figure 2D,E). When sustained oscillations were dampened in some regions (lower *a*_1_/higher *λ*, or higher *a*_2_ and *r*), the tea crop integral (tea yields) was strongly increased (Figure 2C,F). These results suggest that the tritrophic relationship among tea plants, TGLs, and parasitic wasps allows the system to perform cyclic behavior, and that regulation of key parameters can strongly affect tea yields.

### 3.2. Stochastic Simulation Identifies Properties of TGL Outbreaks

**Figure 3:**
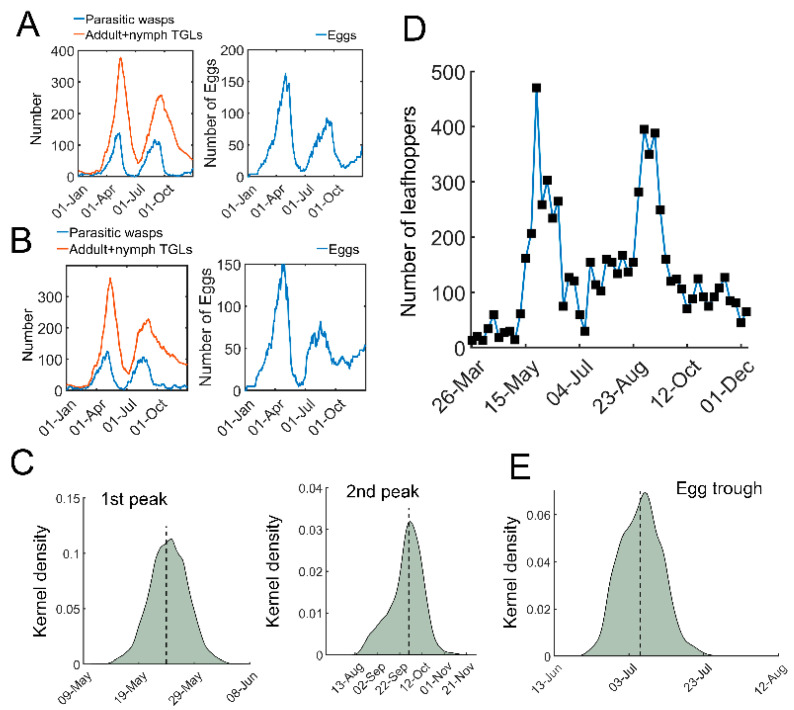
Stochastic simulations of tritrophic interactions. (**A**,**B**) Two representative simulations using stochastic model. Parasitic wasps (blue) and TGLs (adults + nymphs, red) are shown on the left. The number of eggs is shown on the right. (**C**) Kernel density estimation of distribution of the time for the first (left) and the second outbreak (right). (**D**) A field observation of tea green leafhoppers in a tea garden in 2020 at Yangting Village, Wuheng Town, Yixiu District, Anqing City from Southwest Anhui, China. (**E**) Distribution for the trough time of the eggs. The kernel density estimation was based on 5000 sets of stochastic simulations.

We next performed stochastic simulations with temperature-dependent parameters. The daily temperature in 2020 was used as an example (Figure S3). In contrast to the deterministic simulations using ODEs, stochastic trajectories with temperature-dependent regulation indeed showed highly variable dynamics (Figure 3A,B). There were generally two outbreaks of tea green leafhoppers in one year, together with two peaks in the number of eggs (Figure 3A,B). Estimation of the peaks of pest outbreaks showed two skewed distributions (Figure 3C). The median date of the first outbreak was 24 May with 95% confidence interval (CI) [17 May, 31 May], whereas the median for the second outbreak was 30 September [1 September, 29 October] (Figure 3C). In our recent field experiments, two leafhopper outbreaks were identified (Figure 3D). The estimated time for outbreaks in the model was consistent with our field observations (Figure 3C,D) and previous studies [38,39]. In addition, the number of eggs also had a trough, with a median on 6 July with a 95% CI [30 June, 12 July] (Figure 3E), which is compatible with observations [40]. These simulations suggest that our stochastic model is generally consistent with field observations and shows significant variations in temporal trajectories.

### 3.3. Efficiency of Predators Is Higher Compared with Parasitoids in Pest Control

**Figure 4:**
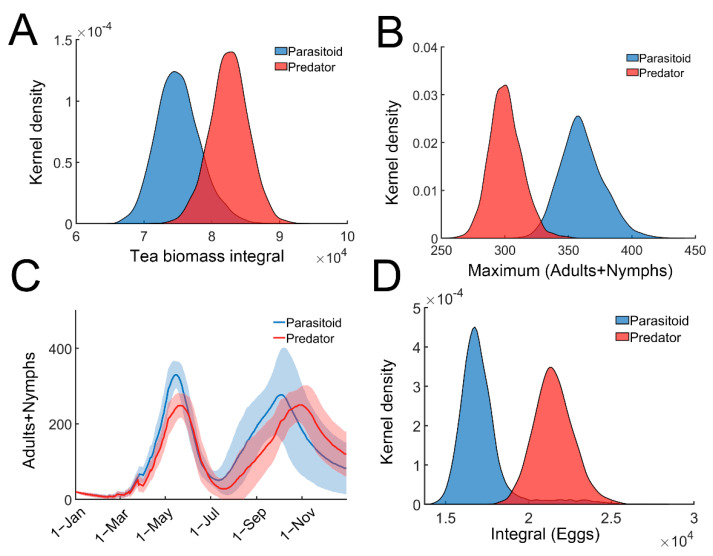
Comparing the efficiency of predators and parasitoids. (**A**) Distribution of the tea crop integral (tea yields) in predator (red) or parasitoid (blue) model. (**B**) Distribution of the maximal TGLs (adults + nymphs) in predator (red) or parasitoid (blue) model. (**C**) Temporal trajectories of maximal TGLs (adults + nymphs) in predator (red) or parasitoid (blue) model. Trajectories were averaged over 2000 simulations with 95% confidence intervals (shaded area). (**D**) Distribution of the temporal integral of eggs in predator (red) or parasitoid (blue) model.

We next compared the efficiency of predation and parasitism for tea pest control. Equation (6) was slightly modified to incorporate the predators (Supplementary materials: Stochastic model for tea plant–TGLs–predator relationship). All parameters remained the same in both the parasitoid and predator models. When only predators were present, the tea crop integral (tea yields) was significantly higher (Figure 4A). Predators were more effective in reducing the leafhopper population (Figure 4B,C). As expected, the egg population recorded in the parasitoid-only model, was remarkably lower than that in the predator-only model (Figure 4D). Since recruited predators feed on adults or nymphs of tea green leafhoppers, the predators can provide direct or instant protection against herbivory attack compared with parasitic wasps, whose protective effect is indirect and delayed. These results suggest that predators may provide better protection against TGL infestation compared with parasitic wasps, and are consistent with field observations [41].

### 3.4. Daily Average Effective Accumulated Temperature Influences the Pest Outbreaks

**Figure 5:**
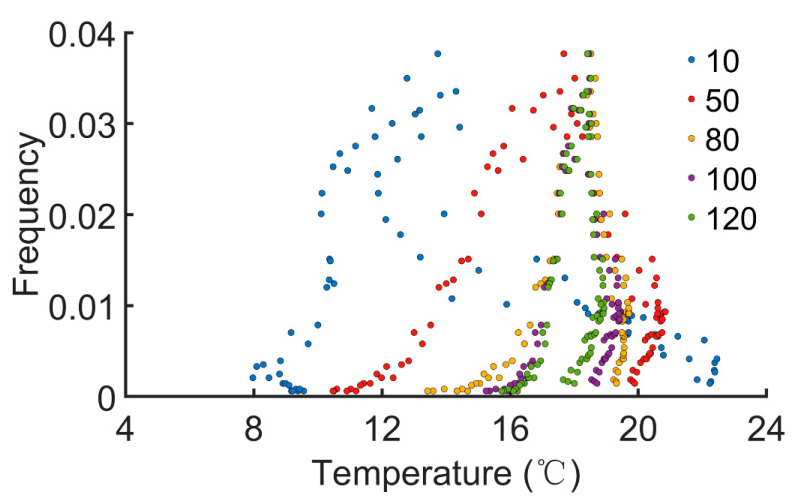
The relation between daily average effective accumulated temperature and pest outbreaks. EAT(°C∙day) is defined as $\sum_{i-n+1}^{i}\left(T_{i}-B\right)$, where *T_i_* is the (average) daily temperature of the *i*th day, and *B* is the baseline or developmental threshold temperature. The duration of record ‘*n*’ is varied from 10, 50, 80, 100, to 120 days.

We next evaluated the effect of temperature on the pest outbreaks. Effective accumulated temperature (°C∙day) is defined as $\sum_{i-n+1}^{i}\left(T_{i}-B\right)$, where *T_i_* is the (average) daily temperature of the *i*th day, *n* is the duration of record, and *B* is the developmental threshold temperature. *B* is set to be 7.8 °C [35]. The daily average EAT is obtained by $\frac{1}{n}\sum_{i-n+1}^{i}\left(T_{i}-B\right)$. The EATs were recorded for various durations (*n* = 10, 50, 80, 100, and 120 days). Then, 5000 stochastic simulations were performed. When pest outbreaks or peaks were identified, the daily average EAT on that day was also calculated. The number of incidences of a pest outbreak or peak on a certain day was normalized to obtain the frequency. We found that occurrence of pest outbreaks was only restricted within narrow ranges of the daily average EAT (roughly 8~23 °C, Figure 5). Daily average EATs outside this range failed to identify a pest outbreak. When the duration of record was longer, the distribution became more concentrated (*n* ≥ 80, Figure 5). These simulations demonstrate that the daily average EAT strongly influences the onset of pest outbreaks.

### 3.5. Slowly Releasing Semiochemicals Increases Tea Yields

**Figure 6:**
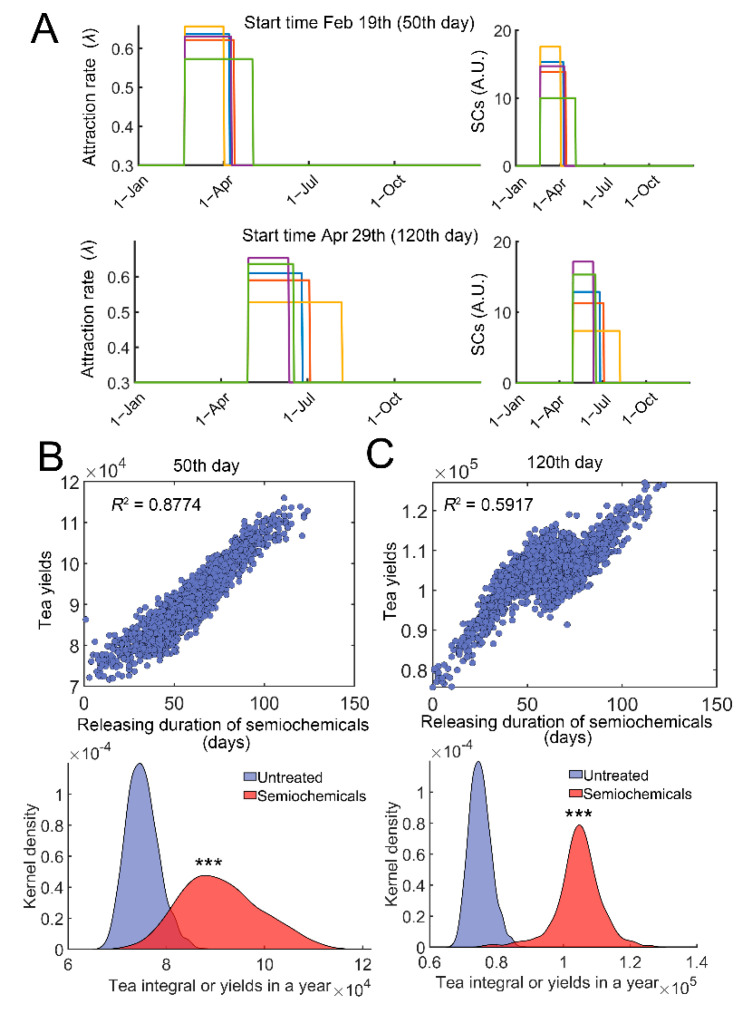
Evaluation of duration of semiochemical releases on tea crop integral (tea yields). (**A**) Examples of variations in attraction efficiency, *λ* and duration of release. *λ* = 0.3(1 + v_max_*SCs/(*SCs* + *K_m_*)), and *SCs* represent the current level of semiochemicals (*v_max_* = 12, and *K_m_* = 12). The start time of semiochemical application was either 19 February (50th day, top) or Apr 29th (120th day, bottom). Five stochastic examples (blue, green, yellow, purple, and red squares) are shown. *λ* variations are shown on the left, and SCs variations are correspondingly provided on the right (curves with the same color). The total amount or dosage of semiochemicals was fixed at 720. The releasing duration was varied following a normal distribution *D*~*N*(60, 20), and *D∙SCs* = 720. (**B**) Correlation of tea yields after using semiochemicals with different durations of releases (Top). The start time was on the 50th day. Distributions of tea yields in the untreated group and group with semiochemical usage (bottom). (**C**) Similar to (**B**), the start time was on the 120th day. In each case, 2000 simulations were performed. ***: *p* < 0.0001.

Pheromones and other semiochemicals are widely used to manage agricultural or forest pests [42]. Synthetic semiochemicals can either be used as repellents or attractants [42]. To simulate the effect of semiochemical application with different releasing rates or durations, we simulated the effect of controlled release. The amount or dosage of semiochemicals was fixed (720 in model 1), whereas the releasing duration was varied (Figure 6A, right). The effect of attractants was evaluated. Attractants can recruit the natural enemies of TGLs [42], analogous to an increase in *λ* values. We modified *λ* = 0.3 (1 + v_max_*SCs/(*SCs* + *K_m_*)); *SCs* represent the current level of semiochemicals. The start time of semiochemical usage was either on 19 February (50th day) or on 29 April (120th day) in the simulations for case studies (Figure 6A). A variable duration of semiochemical release was generated following a normal distribution *D*~*N*(60, 20), where *D* represents duration in days (Figure 6A, right). Since the dosage of semiochemicals was fixed (*D*∙*SCs* = 720), the inner areas of each square were all the same (green, yellow to red, Figure 6A, right). Variations in the durations of release resulted in changes in the efficiency of attracting wasps or parameter *λ* (Figure 6A, left). Stochastic simulations were then performed, and the relation between the tea yields and duration of semiochemical release was determined. Significant correlation was identified irrespective of the starting time (Figure 6B,C, top). Increasing the duration of releases raised the tea yields in a year (Figure 6B,C, bottom panels, *p* < 0.0001). Qualitatively similar results were obtained if semiochemicals were used as repellents (Figure S4).

**Figure 7:**
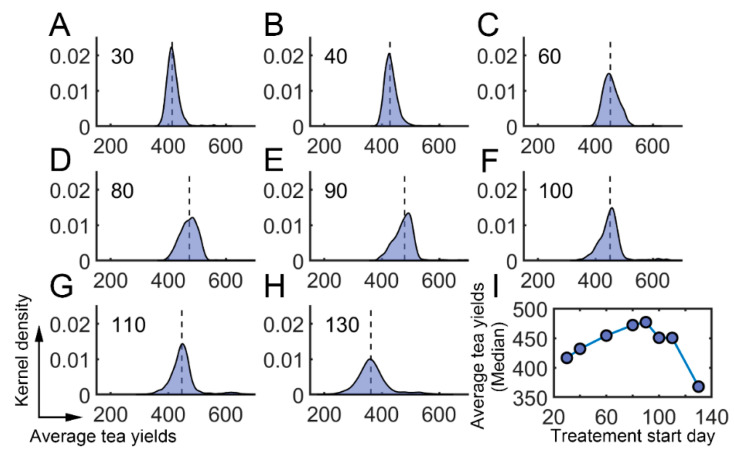
Effect of semiochemicals with different initial application times. (**A**–**H**) The average tea crop integral (tea yields) after applying semiochemicals as attractants on different days. (**A**) 30th day; (**B**) 40th day; (**C**) 60th day; (**D**) 80th day; (**E**) 90th day; (**F**) 100th day; (**G**) 110th day; (**H**) 130th day. (**I**) The median value of average tea crop integral with respect to treatment time.

We further investigated whether there existed an optimum time for the application of semiochemicals. The starting time was varied from the 30th day to the 130th day. The averaged tea yields after using semiochemicals were then determined (Supplementary Materials: Average daily tea yields after semiochemical application). We found that the averaged tea yields first increased and then decreased when the starting time was changed from the 30th day to the 130th day (Figure 7A–H). There was a local maximum around the 90th day (Figure 7I). These results suggest that semiochemicals usage can be optimized to obtain higher average tea yields.

## 4. Discussion

In the current work, we constructed a model based on the tritrophic relationship among tea crops, TGLs, and parasitic wasps, and their interactions can lead to sustained oscillations. We further developed a realistic and stochastic model with temperature-dependent regulation. Generally, stochastic population models perform better than deterministic models in tritrophic ecosystems [43]. Our stochastic simulations showed that two leafhopper outbreaks occur in a year, which is compatible with field observations [44,45]. The first peak was relatively concise, whereas the second one was highly variable (Figure 3C), which is consistent with previous reports [38,39]. Notably, diverse natural enemies such as entomopathogens, specialist and generalist predators coexist in tea ecosystems to exert biological control over tea pests [2]. Tritrophic relationship models can be regarded as interlocked predator–prey models. Even a minimal predator–prey model in tea ecosystems can exhibit complex dynamic patterns, for example, coexistence of multiple limit cycles and saddle node bifurcation [46]. Furthermore, multiple species of herbivores may also compete for a common resource (plant) [14]. Competition also occurs among natural enemies feeding on the same pests. Our model can be more precise if dynamic interactions among multiple predators or parasitoids and natural enemies are considered. Our model does not account for the immigration of leafhoppers, nor of their natural enemies, and these events may be influential on the dynamics of each population.

Previous models have usually ignored the effect of temperature dependence of prey or predator traits, especially in tritrophic interactions involving tea plants and TGLs [45,46,47]. In our current work, the temperature dependency of TGL developmental stages, lifespans, and tea growth rates were incorporated into the stochastic model. We found that effective accumulated temperature plays an important role in TGL outbreaks, with peaks identified within a specific range of average daily EATs (Figure 5). Additionally, the distribution of frequency or possibility of pest outbreaks can be further decreased if the recording duration is increased and becomes relatively stabilized for *n* ≥ 80 (Figure 5). Simulation results indicated that a long-term record of average daily EATs may be essential for predicting TGL outbreaks. If the long-term daily average EAT is above a certain threshold (e.g., approximately 18 °C in our model), the probability of pest outbreaks will be increased, and better pest management strategies should be implemented.

Sensitivity analysis showed that the systemic features are only sensitive to a few parameters (Figure 1D). Specifically, *r*, *K*, and *h* represent the processes responsible for tea plant growth and death or harvesting. Increasing the tea yields by promoting tea growth should not be a priority, as the equilibrium will shift toward higher pest populations via tritrophic relationships. Parameters *μ* (death rate of TGLs) and *ω*_1_ (conversion rate from eggs to nymphs), were both sensitive to temperature [35]. However, the environmental temperature also influences various aspects of ontogenetic processes of leafhoppers and tea plant growth. As demonstrated above, it is not feasible to identify an optimum temperature to maximize tea growth while simultaneously attenuating leafhopper development with the highest efficiency. Furthermore, pesticide application can also decrease pest population (i.e., increasing *μ*) in the short term, but can subsequently result in a resurgence of pests, possibly with higher densities [48]. *c*_1_ is a physiological attribute in TGLs and cannot be easily manipulated. The only possibilities are strategies targeting the insect feeding process (*a*_1_ and *b*_1_) and the attraction of natural enemies (*λ*). The application of synthetic semiochemicals seems to be a feasible way to increase tea yields in tritrophic systems. Repellants can directly decrease insect feeding, whereas attractants, for example, (*E*)-2-hexenal, benzaldehyde, and *α*-farnesene, can be used to lure natural enemies [9,10]. Mass-rearing and field release of parasitoid wasps also represent compelling strategies for integrated pest management by regulating *λ* [49]. All these strategies can indeed lead to a new balance with lower pest populations and provide better biocontrol performance.

With regard to the release of semiochemicals, our simulations suggested that sustained or slow release with low concentrations correlates with higher tea yields (Figure 6). Slow-releasing semiochemicals outcompete fast-releasing regimes, irrespective of the initial application date. Our simulation might explain why volatile semiochemicals used as repellants or attractants should be coated with liquid paraffin to achieve better performance [11]. A possible mechanism is illustrated in Figure S5. When semiochemicals are released in short durations, disruption to pest population will be abated and pests will quickly revert to their original states (outbreak will still occur, Figure S5, red). When semiochemical application lasts longer, pest outbreak will be reduced, with increased tea crop biomass or tea yields (Figure S5, blue). Collectively, using slow-releasing semiochemicals as repellants or attractants might be a compelling strategy in pest control.

## 5. Conclusions

In conclusion, our temperature-dependent stochastic model identified key features of the tritrophic relationship among tea plants, TGLs, and natural enemies in tea ecosystems. We further provided theoretical evidence that slow-releasing semiochemicals targeting insect feeding or the attraction of natural enemies may be highly efficacious. Using more temperature datasets and running the model for consecutive years may provide more insight into the dynamics of different populations in tea ecosystems under future global warming scenarios.

## Data Availability

The data presented in this study are available on request from the corresponding author.

## Supplementary Materials

The following supporting information can be downloaded at: https://www.mdpi.com/article/10.3390/insects13080686/s1. Figure S1: The curve-fitting to experimental measurements; Figure S2: The bifurcation diagram; Figure S3: The daily temperature at the year of 2020; Figure S4: Treatment with volatile semiochemicals to repel TGLs; Figure S5: Semiochemical application with different durations; Table S1: The parameter values and initial conditions; Table S2: Fitted parameters.
