# Supplementary material for: A Temperature-Dependent Model for Tritrophic Interactions Involving Tea Plants, Tea Green Leafhoppers and Natural Enemies
Source: Insects. 2022 Jul 29;13(8):686. doi: 10.3390/insects13080686 (PMC9409375; doi:10.3390/insects13080686)
Supplement: Supplementary file 1 [file insects-13-00686-s001.zip › insects-1813242-supplementary.pdf]

Supplementary materials

Table S1. The parameter values and initial conditions.

Parameters	Values
r	6
K	30
a_1	12
b_1	9
h	1.8
a_2	1.5
b_2	15
δ	4
c_1	0.3
c_2	0.75
μ	0.9
λ	0.3
ω_1	1.5
ω_2	0.9
C	8
P	1
W	0
E	0
N	0

The relationship between temperature and developmental stages

The relationship between temperature and stage durations is described as Arrhenius equation $a \cdot e^{-bT}$ [1], and the fitted parameters were listed in Table S2. The experimental data were adopted from Qiao et al.'s measurements [2]. The fitted curves were shown in Fig.S2. Note that in Table S2, the averaged data for longevity of male and female leafhoppers [2] were fitted. Fitting male and female leafhoppers separately and then acquiring the mean values is also feasible. Simulations are qualitatively the same. We also applied a logistic function to fit the experimental data [2]. However, zeros were included in the confidence intervals for all parameters, suggesting that a logistic type response is not significant and might be inappropriate.

Table S2. Fitted parameters.

Stage duration	a	b [95% CI]	Adjusted R^2
Longevity	60.24 [19.94 100.50]	-0.06648 [-0.1003, -0.0327]	0.9201
Egg	36.46 [25.9 47.02]	-0.06138 [-0.0758, -0.0469]	0.9806
Nymph	58.23 [18.24 98.23]	-0.08121 [-0.1171, -0.0453]	0.9364
Pre-oviposition	10.86 [7.924 13.79]	-0.03159 [-0.0441, -0.0191]	0.9424

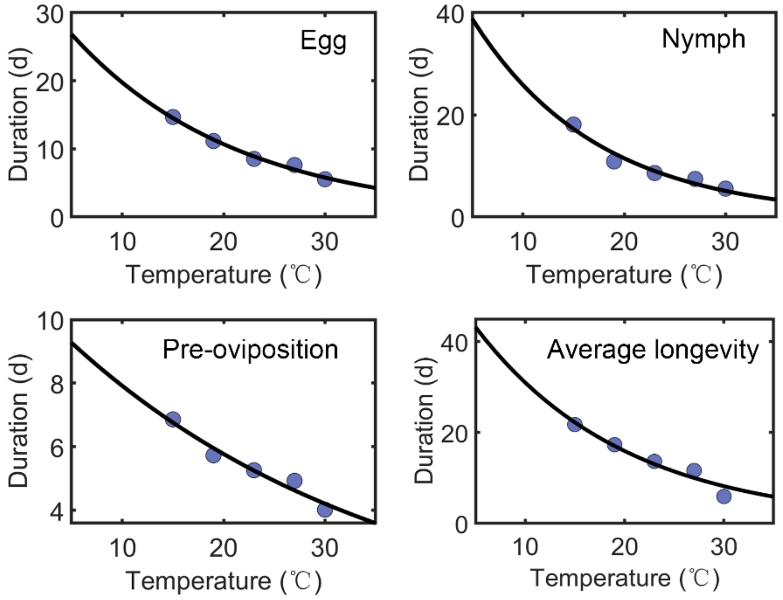

Figure S1. The curve-fitting to experimental measurements. Data were adopted from Qiao et al.'s work [2].

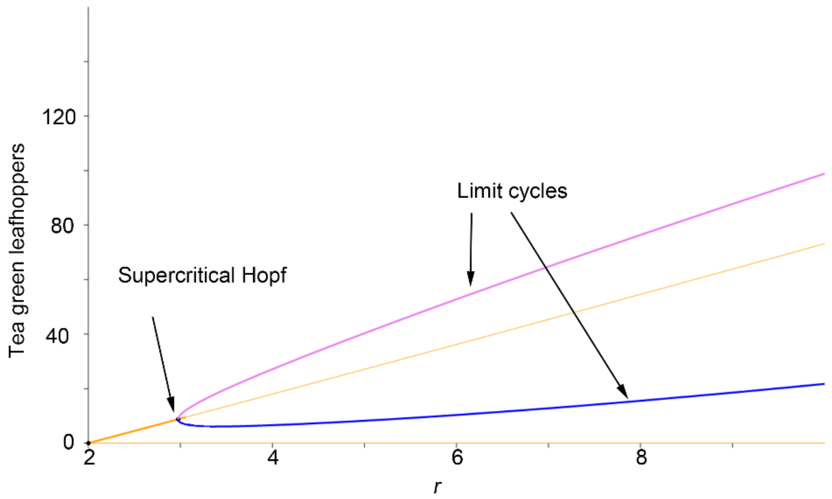

Figure S2. The bifurcation diagram.

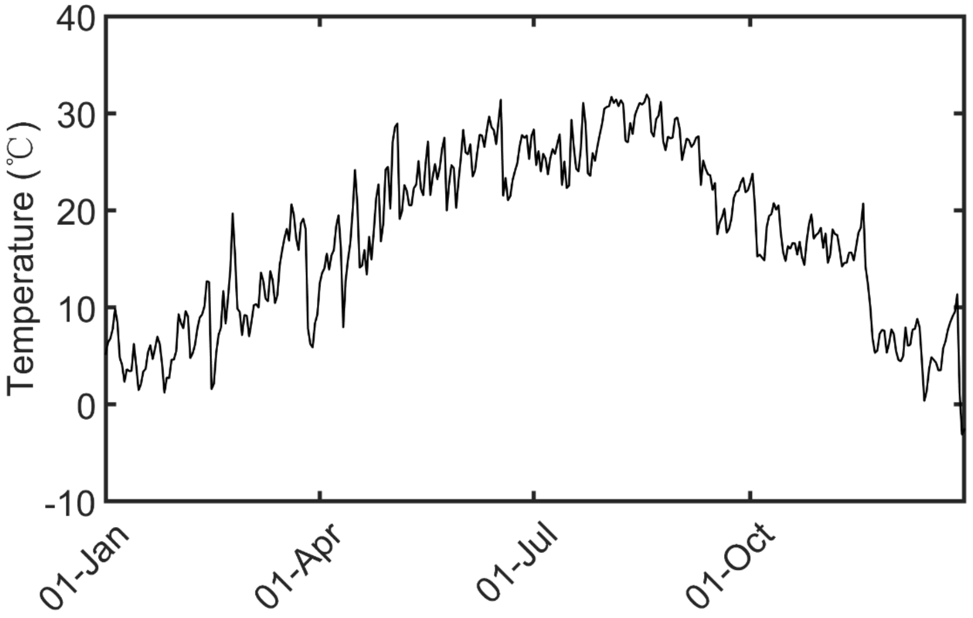

Figure S3. The daily temperature at the year of 2020. Data were adopted from National Oceanic and Atmospheric Administration (http://www.noaa.gov/).

Stochastic model for tea plant-TGLs-predator relationship

To compare the effect of predators and parasitoids, we modified our stochastic model. The predators (e.g. spiders, dominant arthropods in tea grown regions) can feed on both the adults and nymphs of tea green leafhoppers [3]. The model was modified as follows:

$$\frac{dC}{dt} = r(T)C(1-\frac{C}{K}) - \frac{a_1 C(P+N)}{b_1+C} - hC$$

$$\frac{dP}{dt} = \psi_2\omega_2(T)N - \mu(T)P - \frac{a_2 P(fS)}{b_2+P}$$

$$\frac{dS}{dt} = \frac{c_2 a_2 N(1-f)S}{b_2+N} + \frac{c_2 a_2 P(fS)}{b_2+P} + \frac{\lambda a_1 C(P+N)}{b_1+C} - \delta S$$

$$\frac{dE}{dt} = \frac{c_1 a_1 CP}{b_1+C} - \psi_1\omega_1(T)E$$

$$\frac{dN}{dt} = \psi_1\omega_1(T)E - \psi_2\omega_2(T)N - \frac{a_2 N(1-f)S}{b_2+N}$$

where f is the fraction of predators (e.g. spiders) which feeds on adult tea green leafhoppers. We assumed that f is binomially distributed $f \sim B(1, 0.5)$. This equation was used for stochastic simulations using τ-leap method [4]. All other parameters and initial conditions were held the same as stochastic model for parasitic wasps.

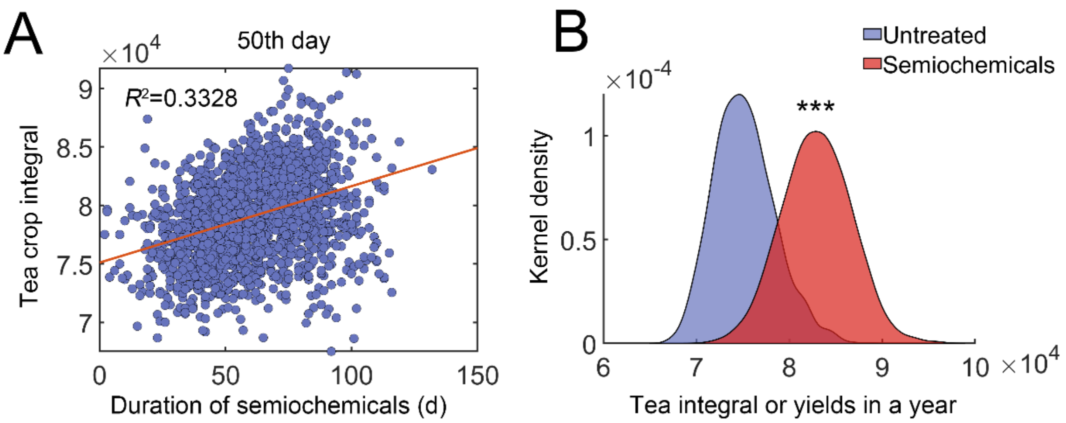

Figure S4. Treatment with volatile semiochemicals to repel TGLs. (A) The total amount of semiochemicals was fixed at 720 and the releasing rate (or duration of semiochemical release) was varied. (B) The distribution of tea crop integral (tea yields or temporal integration of tea crop biomass) in untreated and semiochemical-treated groups. ***: $P < 0.0001$. The treatment start time is at 50th day from the beginning of the year. Using semiochemicals as repellents to decrease insect feeding or a_1 values, $a_1 = 12/(1+SCs/K_m)$, where $K_m = 2$. SCs is the level of semiochemicals. The duration $D \sim N(60, 20)$, and $D \cdot SCs = 720$.

Average daily tea yields after semiochemical application

The integrated tea crop (tea yields) after semiochemical application is defined as:

$$I = \int_{T_0}^{T_{end}} C \cdot \mathrm{d}t$$

where C stands for tea crop biomass, T_0 is the time of semiochemical application, T_{end} is the end of the year. Then, the average daily tea yields (the average level in tea crop integration after using semiochemicals) can be defined as:

$$I_{ave} = \frac{1}{T_{end} - T_0} \int_{T_0}^{T_{end}} C \cdot \mathrm{d}t$$

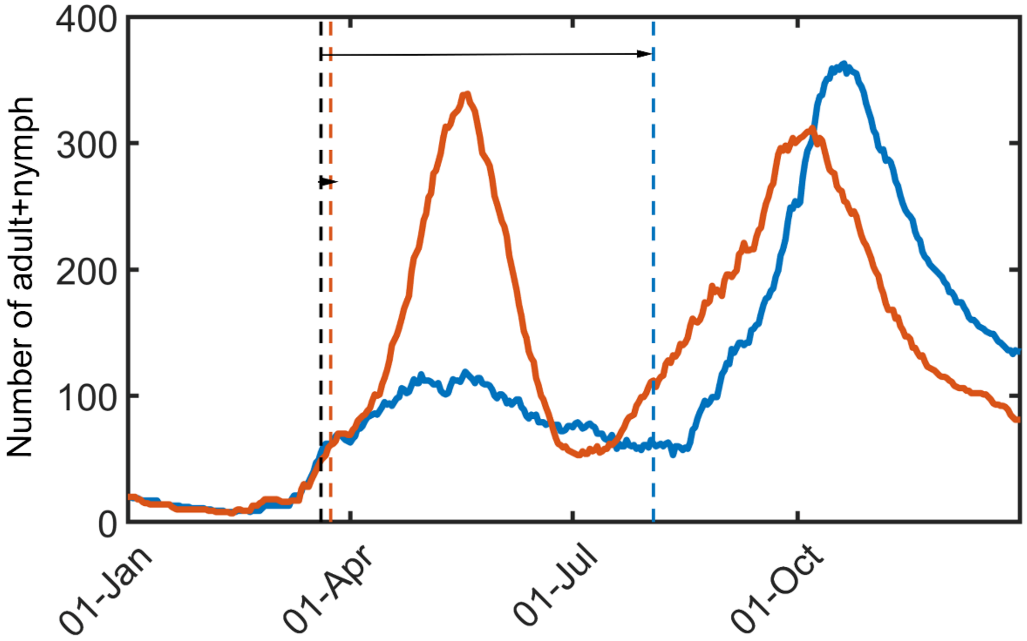

Figure S5. Semiochemical application with different durations. The start of semiochemical treatment is 80th day. The black dashed vertical line denotes the start of semiochemical usage. The red dashed vertical line represents a duration of semiochemical release for 4 days. The red solid curve showed two pest peaks. The blue dashed line represents a duration of semiochemical release for 136 days. The blue solid curve showed only one peak.

References

1. Davidson, A.T.; Hamman, E.A.; McCoy, M.W.; Vonesh, J.R. Asymmetrical effects of temperature on stage-structured predator–prey interactions. *Functional Ecology* **2021**, *35*, 1041-1054.
2. Qiao, L. Response of Empoasca onukii Matsuda to Short-Term High or Low Temperature and the Molecular Mechanisms; Northwest A & F University: Xianyang, China, 2015.
3. Ye, G.-Y.; Xiao, Q.; Chen, M.; Chen, X.-x.; Yuan, Z.-j.; Stanley, D.W.; Hu, C. Tea: biological control of insect and mite pests in China. *Biological Control* **2014**, *68*, 73-91.
4. Tian, T.; Burrage, K. Stochastic models for regulatory networks of the genetic toggle switch. *Proc Natl Acad Sci U S A* **2006**, *103*, 8372-8377.

The MATLAB code for deterministic equations ‘CPNdefault.m’

```
function out=CPNdefault(t,y)
global par;
r=par(1);
K=par(2);
a1=par(3);
b1=par(4);
h=par(5);
c1=par(6);
a2=par(7);
b2=par(8);
mu=par(9);
c2=par(10);
delta=par(11);
c3=par(12);
mu2=par(13);
mu3=par(14);
v1=r*y(1)*(1-y(1)/K);
v2=a1*y(1)*(y(2)+y(5))/(b1+y(1));
v3=h*y(1);
v4=a2*y(4)*y(3)/(b2+y(4));
v5=mu*y(2);
v6=delta*y(3);
v7=mu2*y(4);
v8=mu3*y(5);
v9=a1*y(1)*(y(2))/(b1+y(1));
    dxdt=v1-v2-v3; %Tea y(1)
    dydt=v8-v5;% Pest addults TGL y(2)
    dzdt=c2*v4+c3*v2-v6; % Natural enemies y(3)
    dedt=c1*v9-v7-v4;% Eggs y(4)
    dndt=v7-v8;% Nymphs y(5)
    out=[dxdt;dydt;dzdt;dedt;dndt];
end
```

The MATALB code for simulating the deterministic equations. Note that the code below must be placed in the same folder with ‘CPNdefault.m’

```
global par
hold on;
V=20; %scale parameter from concentration to numbers
par=[6,  30*V,   12,    9*V,  1.8,  0.3,  1.5, 15*V,  0.9,  0.75, 4,  0.3  1.5   0.9];
t0=[0:0.02:96];
y0=[8,  1    0  0   0]*V;
for i=4:2:12
   par(1)=i;
[t,y]=ode23s(@CPNdefault,t0,y0);
cols=[0.2 0.25 0.74;
     0.85 0.33 0.1;
     0.93 0.69 0.13;
     0.49 0.18 0.56;
     0.47 0.67 0.19;
     0.30 0.75 0.93;
     0.64 0.08 0.18];
vec=2001:length(t0);
figure(1);
plot3(y(vec,1),y(vec,2)+y(vec,5),y(vec,3),'color',cols(1,:).*[1 1 (i-
2)/8],'linewidth',4);
hold on;
end
set(gca,'color','w','xtick',0:200:600,'ytick',0:200:400,'ztick',0:50:150,'fontsize',40
,'linewidth',4);
set(gcf,'color','w');
xlim([-50 600]);
ylim([0 360]);
zlim([0 150]);
axis square;
box off;
view([-52 40]);
```

The basic framework for stochastic simulations:

```
global T
T=1/30;
% daily averaged temperature from 2020
Tdays=[5.1250,6.387500,6.787500,7.762500,9.850000,8.462500,4.837500,4.062500,2.325000,
3.625000,3.440000,3.45714285714286,6.250000,3.987500,1.475000,2.087500,3.387500,3.6625
00,5.387500,6.100000,4.675000,5.775000,6.987500,6.225000,4.150000,1.212500,2.775000,2.
700000,4.600000,4.625000,5.487500,9.300000,8.400000,7.850000,9.62857142857143,9.062500
,4.775000,5.262500,6.162500,7.825000,...
8.950000,9.262500,10.10000,12.72500,12.58750,1.575000,2.125000,5.312500,7.162500,7.900
000,11.70000,8.312500,10.91250,13.86250,19.68750,15.37500,9.875000,9.525000,7.137500,9
.212500,9.137500,7.012500,8.537500,10.22500,10.32500,9.987500,13.60000,12.72500,10.900
00,10.63750,13.75000,12.78750,10.43750,11.27500,14.36250,15.83750,17.12500,18.10000,16
.85000,20.63750,...
19.52500,17.11250,15.90000,18.65000,19.13750,18.08750,7.825000,6.237500,5.875000,8.387
500,9.262500,12.46250,13.53750,14.02500,15.55000,13.88750,15.3571428571429,15.91250,18
.35000,19.50000,16.03750,7.937500,12.70000,14.90000,16.63750,19.92500,24.17500,20.5875
0,14.10000,14.31250,15.92500,13.37500,17.28750,14.92500,17.91250,21.23750,22.68750,16.
80000,18.60000,24.18750,...
24.48750,20.16250,27.03750,28.57500,28.95000,19.10000,19.93750,22.60000,22.01250,20.53
750,20.51250,22.27500,22.65000,25.08750,22.22500,21.55000,24.75000,27.10000,21.56250,2
3.42500,24.77500,23.20000,24.33750,26.32500,27.50000,19.97500,22.77500,24.65000,24.350
00,20.23750,23.03750,25.41250,28.30000,25.97500,25.76250,26.8142857142857,23.48750,24.
08750,26.03750,27.80000,...
27.68750,26.52500,28.21250,29.67500,28.57500,28.26250,26.81250,29.17500,31.41250,21.50
000,23.35000,21.05000,21.46250,23.13750,24.05000,24.87500,26.63750,27.76250,27.46250,2
7.70000,25.27500,27.65000,28.33750,24.65000,26.11250,24.00000,25.82500,25.33750,23.700
00,25.36250,26.31250,25.81250,26.86250,27.83750,22.60000,25.03750,22.30000,22.56250,29
.33750,26.51250,24.30000,24.01250,26.38750,31.08750,28.87500,23.81250,23.55000,25.9000
0,25.10000,26.53750,27.77500,28.90000,30.47500,30.65000,30.73750,31.68750,...
31.06250,31.41250,30.73750,31.35000,30.93750,27.16250,27,29,27.85000,29.80000,30.47500
,31.07500,30.90000,31.15000,31.92500,31.46250,28.06250,27.60000,29.40000,29.75000,31.1
8750,27.12500,26.18750,27.60000,27.42500,27.48750,29.41250,29.57500,28.37500,25.16250,
26.28750,27.41250,27.20000,26.53750,26.86250,27.47500,27.63750,22.60000,25.13750,24.30
000,23.72500,23.60000,22.10000,22.82500,17.52500,18.73750,19.28750,20.17500,17.70000,1
8.10000,19.16250,21.26250,21.88750,22.02500,22.86250,23.35000,21.86250,22.07500,...
22.83750,23.78750,20.28750,15.23750,15.43750,15.13750,14.83750,18.27500,19.41250,19.61
250,20.75000,20.07500,20.51250,17.63750,15.66250,14.77500,16.31250,16.03750,16.63750,1
6.58750,15.42500,16.76250,15.18750,14.36250,16.86250,18.60000,19.58750,17.08750,17.462
50,17.67500,18.21250,16.12500,17.62500,14.58750,15.42500,18.0428571428571,17.55000,17.
45000,15.95000,14.21250,14.58750,14.60000,15.63750,15.65000,14.83750,16.25000,17.70000
,18.21250,20.71250,14.20000,12.42500,10.11250,6.800000,5.325000,5.537500,7.262500,...
7.675000,7.600000,5.325000,6.300000,7.737500,7.262500,5.475000,4.587500,4.450000,4.987
500,7.950000,6.050000,6.150000,7.712500,7.787500,8.825000,7.912500,4.300000,0.3625000,
```

1.412500,3.600000,4.862500,4.600000,4.287500,3.512500,3.537500,5.775000,6.525000,7.687500,8.462500,9.062500,9.550000,11.36250,1.212500,-3.100000,-2.612500,0.7875000,3.275000,1.575000,6.475000,3.950000,2.225000,-4.362500,-3.425000,-0.1625000,2.075000,2.162500,6.600000,9.437500,11.65000,10.10000,2.925000,3.350000,5.362500,7.275000,10.38750,...

8.712500,7.462500,6.625000,7.650000,6.550000,6.175000,6.525000,6.012500,6.912500,9.725000,8.887500,7.500000,6.737500,10.45000,8.975000,7.487500,9.475000,11.13750,6.325000,8.31428571428572,10.45000,5.225000,0,13.10000,14.82500,10.97500,11.72500,8.500000,8.925000,14.55000,14.3333333333333,17.82000,17.23750,11.72500,11.58750,7.412500,6.200000,8.312500,10.78750,7.300000,7.862500,8.025000,10.78750,10.47500,7.562500,6.187500,7.637500,10.31250,11.68750,10.97500,11.63750,12.65000,16.01250,17.17500,12.47500,9.200000,10.26250,9.237500,...

9.887500,11.21250,10.35000,13.80000,14.48750,17.06250,19.13750,15.43750,17.36250,20.45000,15.96250,14.17500,13.63750,13.85000,12.35000,13.03750,12.51250,16.28750,11.33750,12.18750,15.96250,16.66250,14.50000,14.50000,17.55000,13.31250,16.67500,18.97500,17.78750,15.52500,18.46250,21.60000,19.56250,18.28750,18.48750,16.70000,15.16250,15.85000,17.03750,19.07500,22.22500,24.63750,21.83750,19.95000,23.03750,19.32500,21,18.77500,22.52500,25.08750,26.53750,25.47500,20.02500,21.18750,23.67500,25.53750,24.88750,16.42500,...

18.18750,19.41250,18.15000,19.75000,22.88750,23.97500,21.16250,24.25000,24.83750,20.72500,23.78750,23.53750,24.60000,25.57500,27.03750,27.12500,27.56250,21.35000,23.06250,24.86250,26.30000,27.62500,27.85000,29.0857142857143,25.53750,27.73750,27.61250,26.27500,30.23750,30.51250,29.07500,26.97500,21.82500,21.05000,22.42500,24.81250,26.06250,27.33750,28.10000,26.95000,28.01250,25.48750,27.53750,28.77500,27.68750,26.31250,26.22500,24.67500,24.42500,27.71250,30.28750,29.02500,26.80000,29.20000,29.63750,31.06250,30.92500,31.43750,...

31.48750,32.12500,30.62500,24.05000,25.75000,28.56250,30.35000,30.10000,29.27500,28.83750,26.73750,27.78750,26.40000,26.15000,27.53750,28.30000,29.45000,29.26250,30.65000,30.48750,29.76250,29.75000,29.91250,29.73750,29.72500,29.67500,28.05000,26.61250,26.36250,26.20000,22.95000,23.01250,24.11250,25.73750,26.82500,28.05000,28.65000,28.73750,27.02500,29.30000,28.61250,24.26250,26.07500,24.62500,23.43750,26.58750,28.26250,30.11250,30.30000,29.26250,26.30000,27.71250,28.85000,26.05000,24.51250,23.80000,25.76250,27.32500,28.26250,...

28.40000,27.08750,26.10000,24.85000,24.92500,25.7285714285714,25.51250,26.91250,29.48750,22.47500,26.67500,26.51250,27.91250,28.43750,28.61250,29.15000,25.86250,27.55000,22.01250,23.53750,24.47500,26.10000,28.61250,28.08750,22.50000,21.62500,18.05000,18.78750,21.82500,18.25000,16.18750,19.26250,18.08750,19.47500,16.27500,12.97500,12.02500,12.5333333333333,14.68750,10.32500,11.62500,13.25000,16,15.85000,15.40000,17.68750,18.20000,17.23750,17.23750,17.40000,19.06250,19.25000,16.82500,17.43750,18.50000,17.85000,19.07500,7,6.462500,9.500000,11.27500,...

13.96250,13.37500,13.16250,13.13750,15.91250,16.08750,13.10000,11.43750,13.15000,15.71250,11.40000,5.762500,6.575000,10.67500,11.28750,12.53750,11.40000,14.9285714285714,14.56250,9,5.087500,6.925000,8.412500,9.350000,9.500000,11.52500,10.47500,11.30000,11.45000,12.22500,10.13750,7.825000,7.512500,11.30000,11.67500,10.57500,5.762500,4.387500,8

```
.175000,11.60000,9.975000,10.16250,11.85000,4.925000,-0.8125000,-
0.7500000,1.100000,4.08571428571429,5.200000,5.425000,6.912500];
V=20;
Scale=1;
r=6*Scale; K=30*V; a1=12*Scale; b1=9*V; h=1.8*Scale; c1=0.3;
a2=1.5*Scale; b2=15*V; mu=0.9*Scale; c2=0.75; delta=4*Scale; c3=0.3;
N=366+365;
ti=1:N;
Nrep=1; % Replicating number of stochastic simulations
flag=2.5;
Crop=zeros(N,Nrep);
Pest=zeros(N,Nrep);
Nat=zeros(N,Nrep);
Eggs=zeros(N,Nrep);
Nymphs=zeros(N,Nrep);
%       Tea  TGL  NE   Eggs Nymphs
y0=round([8    1    0     0    0]*V);
Crop(1,:)=y0(1).*ones(1,Nrep);
Pest(1,:)=y0(2).*ones(1,Nrep);
Nat(1,:)=y0(3).*ones(1,Nrep);
Eggs(1,:)=y0(4).*ones(1,Nrep);
Nymphs(1,:)=y0(5).*ones(1,Nrep);
Tcrit2=11;
Tcrit3=8;
for j=1:Nrep
for i=1:(N-1)
   mu=2/(LifeTime2(i,1,Tdays)+LifeTime2(i,2,Tdays))*30*(1+0.1*(2*rand-1));
   days1=round(Duration2(i,1,Tdays));
   mu2=1/days1*30*(1+0.1*(2*rand-1));
   days2=round(Duration2(i,2,Tdays)+Duration2(i,3,Tdays));
   mu3=1/days2*30*(1+0.1*(2*rand-1));
   Beta=20;
   GrowthCoeff=exp(-((Tdays(i)-23)/Beta)^2);

      if Tdays(i)<=Tcrit2
         flag2=0;
      else
         flag2=1;
      end

      if Tdays(i)<=Tcrit3
         flag3=0;
      else
         flag3=1;
```

```
    end

r=6*GrowthCoeff+3;
v1=r*Crop(i,j)*(1-Crop(i,j)/K);
v2=a1*Crop(i,j)*(Pest(i,j)+Nymphs(i,j))/(b1+Crop(i,j));
v3=h*Crop(i,j);
v4=a2*Pest(i,j)*Nat(i,j)/(b2+Pest(i,j));
v5=mu*Pest(i,j);
v6=delta*Nat(i,j);
v7=mu2*Eggs(i,j);
v8=mu3*Nymphs(i,j);
v9=a1*Crop(i,j)*(Pest(i,j))/(b1+Crop(i,j));

f1=poissrnd(v1*T);
f2=poissrnd(v2*T);
f3=poissrnd(v3*T);
f4=poissrnd(v4*T);
f5=poissrnd(v5*T);
f6=poissrnd(v6*T);
f7=poissrnd(v7*T);
f8=poissrnd(v8*T);
f9=poissrnd(v9*T);

Crop(i+1,j)=Crop(i,j)+f1-(f2+f3);
Pest(i+1,j)=Pest(i,j)+flag3*f8-f5;
Nat(i+1,j)=Nat(i,j)+round(c2*f4+c3*f2)-f6;
Eggs(i+1,j)=Eggs(i,j)+flag*(ceil(c1*f9))-flag2*f7-f4;
Nymphs(i+1,j)=Nymphs(i,j)+flag2*f7-flag3*f8;
 % control for negative population
if Pest(i+1,j)<=0
    Pest(i+1,j)=0;
end
if Crop(i+1,j)<=1
    Crop(i+1,j)=1;
end
if Nat(i+1,j)<=0
    Nat(i+1,j)=0;
end
if Eggs(i+1,j)<=0
    Eggs(i+1,j)=0;
end
if Nymphs(i+1,j)<=0
    Nymphs(i+1,j)=0;
end
```

```
end
CPN=[Crop Pest+Nymphs Nat];
figure(1);
plot(1:731,CPN(:,2),'linewidth',2);
hold on;
xlim([1 366]);
set(gcf,'color','w');
xtickangle(45);
ylabel('TGL+Nymphs');
end

function out=LifeTime2(ti,flag,temperature)
if ti>365+366
   ti=ti-365-366;
end
temp=temperature(ti);
% using averaged lifespan of males + females are also applicable as in Qiao et al.
work
for i=1:length(flag)
if flag(i)==1 % female
   out(i)=66.79.*exp(-0.06624.*temp);
elseif flag(i)==2 % male
   out(i)=55.02.*exp(-0.06695.*temp);
end
end
end

function out=Duration2(ti,flag,temperature)
if ti>365+366
   ti=ti-365-366;
end
temp=temperature(ti);
if flag==1 % eggs
   out=36.46.*exp(-0.06138.*temp);
elseif flag==2 % Nymph
   out=58.23.*exp(-0.08121.*temp);
elseif flag==3 % preoviposition
   out=10.86.*exp(-0.03159.*temp);
else
   disp(['Flag error']);
end
end
```
